# Establishment and experimental validation of a novel cuproptosis-related gene signature for prognostic implication in cholangiocarcinoma

**DOI:** 10.3389/fonc.2022.1054063

**Published:** 2022-12-08

**Authors:** Jialu Chen, Xiaopeng Yu, Huanjun Tong, Chengwei Tang, Zhaohui Tang

**Affiliations:** Department of General Surgery, Xinhua Hospital, Shanghai Jiao Tong University, School of Medicine, Shanghai, China

**Keywords:** Cuproptosis, cholangiocarcinoma, prognosis, tumor immune microenvironment, lipid metabolism

## Abstract

**Background:**

Cholangiocarcinoma (CCA) is a highly malignant, heterogeneous bile duct malignancy with poor treatment options. A novel type of cell death termed cuproptosis was recently demonstrated to closely correlate with tumor progression. To gain more insight into the role of cuproptosis in CCA, we investigated the prognostic implications of cuproptosis related genes (CRGs) and their relationship to the development of CCA.

**Methods:**

Gene expression data for CCA were obtained from the European Bioinformatics Institute (EMBL-EBI) database. Least absolute shrinkage and selection operator (LASSO) penalized Cox regression was used to construct a prognostic risk model based on CRGs. RNA-seq, qRT−PCR and immunohistochemistry staining were used to verify the expression of CRGs in human CCA tissues or cell lines. Further *in vitro* experiments were performed to demonstrate the role of cuproptosis in CCA.

**Results:**

We established a 4-gene signature (ATP7A, FDX1, DBT and LIAS) that exhibited good stability and was an independent prognostic factor for CCA. Seventy-five CCA samples were divided into high- and low-risk groups based on the risk score. Enrichment analysis revealed increased extracellular activity in the high-risk group and increased lipid metabolic activity in the low-risk group. Moreover, the 4 signature genes were verified in clinical samples and cell lines by RNA-seq, qRT−PCR and immunohistochemistry. Further experiments confirmed that cuproptosis can significantly inhibit the viability of CCA cells. Knockdown of the key gene LIAS ameliorated the toxicity of cuproptosis to CCA cells.

**Conclusion:**

We established a 4-gene prognostic signature based on cuproptosis and explored the role of cuproptosis in CCA. The results provide an effective indicator for predicting the prognosis of cuproptosis in CCA.

## Introduction

Cholangiocarcinoma (CCA) is a highly heterogeneous hepatobiliary malignancy arising through malignant transformation of cholangiocytes (1). The overall incidence of CCA is increasing worldwide. By anatomical location, CCA is divided into three different types: intrahepatic cholangiocarcinoma (iCCA), perihilar cholangiocarcinoma (pCCA), and distal cholangiocarcinoma (dCCA) (2). The onset of CCA is insidious and often asymptomatic at first diagnosis. It is highly invasive and can invade perihepatic tissues and lymph nodes and generate distant metastases at an early stage. Radical resection is the only way to cure CCA, but due to its insidious onset, the best time for surgery has often passed when CCA is diagnosed. The postoperative recurrence rate of CCA is still as high as 40%-80%. The 5-year survival rate for CCA is approximately 10% (3) and the R0 resection rate is <30% (4). Therefore, further scientific insights are needed to improve patient outcomes related to this highly lethal disease.

Previously, different modes of cell death have attracted much attention. The potential of apoptosis, ferroptosis, pyroptosis and necroptosis in cancer treatment has been widely explored. Previous studies have found that various metal ions can trigger cell death in different ways. Such as ferroptosis caused by the peroxidation of phospholipids, and pyroptosis performed by inflammasomes and gasdermin proteins (5, 6). Cuproptosis is a recently discovered novel type of cell death that is distinct from known cell death processes such as ferroptosis and pyproptosis (7). Copper is an essential trace metal for protein function in cells (8, 9). The right amount of copper can meet the needs of the body, while too much copper can induce cell death (10). After intracellular copper ions accumulate, they can bind to thioredoxin and induce its oligomerization and abnormal aggregation (11) or reduce the level of Fe-S cluster proteins, both of which lead to a proteotoxic stress response and eventually induce cell death, i.e., cuproptosis. Cuproptosis is closely associated with the tricarboxylic acid (TCA) cycle. Mitochondrial respiration-dependent cells are more sensitive to cuproptosis (7). Key genes include FDX1, LIAS, and other genes encoding lipoic acid pathway-related enzymes (12). Copper promotes oligomerization of DLAT, which in turn promotes an increase in insoluble DLAT, leading to proteotoxic stress and cell death.

Current studies suggest that cuproptosis plays a significant role in the development of tumors and antitumor processes (13, 14). Nevertheless, its specific function in CCA has not been validated. Therefore, we systematically investigated the prognostic implications of cuproptosis-related genes (CRGs) in CCA as well as their expression levels and biological functions and studied the intrinsic link between cuproptosis and the tumor immune microenvironment to better understand the role of cuproptosis in the prognosis and progression of CCA.

## Materials and methods

### Data acquisition and preprocessing

The E-MTAB-6389 dataset was downloaded from the European Bioinformatics Institute (EMBL-EBI) database (https://www.ebi.ac.uk/). The dataset information was as follows: expression profiles acquired by microarray, overall survival (OS), sex, and other clinical indicators. We excluded samples with missing OS or OS less than or equal to 1 month, leaving 75 tumor samples and 29 normal samples for follow-up analysis. Genes with missing values were excluded by the Limma package (15) in R (4.1.3). For the case of a multiprobe corresponding to a gene, the median value was taken as its final expression value.

### Clinical patients and CCA tissues

Thirty-eight paired paracancerous and tumor tissues were collected from CCA patients who underwent radical cystectomy in the Xinhua Hospital Affiliated to Shanghai Jiao Tong University School of Medicine. (Shanghai, China). All patients were diagnosed with CCA according to the World Health Organization (WHO) criteria. The ethics committee of Xinhua Hospital approved this study.

### Construction of a CRG-based CCA prognostic model

A total of 13 genes strongly linked to cuproptosis were extracted from a previous study. On the basis of the expression levels of CRGs and OS of included patients, univariate Cox regression was performed to analyze the correlation between CRGs and survival status, and the E-MTAB-6389 cohort candidate key genes were screened. LASSO Cox regression was performed to reduce overfitting (10-fold cross-validation, P value = 0.05 per 1000 cycles). The final risk signature was built and calculated by the coefficients of the 4 CRGs and their expression levels. Riskscore =∑ (Xi × Yi) (X: coefficient, Y: gene expression) 0.7958*ATP7A+(-0.1946) *DBT+(-0.6824) *FDX1+(-0.9715) *LIAS. On the basis of the value of the median risk score, patients were divided into high-risk and low-risk groups. Survival outcomes were assessed by Kaplan−Meier (KM) analysis for both subgroups. In addition, principal component analysis (PCA) on the basis of 4 genetic characteristics was performed with the “t-SNE” R package. The R packages “survival”, “survminer” and “timeROC” were utilized for 1-, 3- and 5-year receiver operating characteristic (ROC) curves.

### Independent prognostic analysis of the risk score

Clinical information of the patients in the E-MTAB-6389 cohort was extracted and analyzed in our regression models in combination with the risk score. Univariate and multivariate Cox regression models were used to assess whether the risk model was an independent prognostic factor.

### Functional enrichment analysis based on the risk score

CCA patients in the E-MTAB-6389 cohort were divided into two groups according to the median value of the risk scores. The Limma package in R (4.1.3) was used to analyze the differences between the two risk groups. Differentially expressed genes between the two groups were screened according to specific criteria (FDR < 0.05). Gene Ontology (GO) and Kyoto Encyclopedia of Genes and Genomes (KEGG) analyses were performed with the “clusterProfiler” package (16).

### Gene Set Enrichment Analysis (GSEA)

We applied GSEA to investigate further molecular mechanisms (17). The gene set “c2.cp.v7.2.symbols” was acquired from the MSigDB database (18). An adjusted p value < 0.05 and FDR < 0.25 were deemed to be statistically significant. Enrichment analysis was performed using the R package “clusterProfiler” (16).

### Tumor immune microenvironment analysis

The abundance of 22 immune cells in the high- and low-risk groups was compared via CIBERSORTx (https://cibersortx.stanford.edu/). Single sample gene set enrichment analysis (ssGSEA) was utilized to assess the correlation between the abundance of infiltrating immune cells and the risk score.

### Cell culture and transfection

The immortalized human normal bile duct epithelial cell line H69 and CCA cell lines RBE, HCCC-9810, QBC-939, CCLP-1 and HUCCT-1 were purchased from the Cell Resource Centre of Shanghai Institutes for Biological Sciences. The cell lines were authenticated using short tandem repeat analysis. The H69, RBE, HCCC-9810, CCLP-1 and HUCCT-1 cells were cultured in Roswell Park Memorial Institute 1640 (RPMI-1640) medium (Gibco, USA), and the QBC-939 cells was maintained in Dulbecco’s modified Eagle medium (DMEM) (Gibco). Both media were supplemented with 10% fetal bovine serum. All cells were grown at 37°C with 21% O2 and 5% CO2.

### RNA-seq of CCA tissues

Tumorous and paracancerous tissues were collected from 7 patients who underwent curative surgery for CCA at Xinhua Hospital Affiliated with Shanghai Jiao Tong University School of Medicine. Total RNA of 14 samples was first extracted and tested for RNA quality. Then, cDNA was synthesized using the interrupted RNA as a template. The purified cDNA was then A-tail added, and the sequencing junction was ligated. Finally, PCR amplification was performed. The constructed RNA library was quality checked with an Agilent 2100 Bioanalyzer and then sequenced with an Illumina sequencer. The raw image data files obtained from high-throughput sequencing were transformed into raw data with base calling analysis, and the results were stored in FASTQ file format. Trimmomatic software was used to preprocess the raw data for quality and to statistically summarize the number of reads throughout the quality control process. Hisat2 was used to sequence the clean reads against a specified reference genome. The expression level of protein-coding genes was estimated by reads localized to the genomic region of the protein-coding genes or to the exonic region of the protein-coding genes. The htseq-count software was used to obtain the number of reads that matched the protein-coding genes in each sample. Cufflinks software was used to calculate the FPKM values for the expression of protein-coding genes.

### RNA isolation and quantitative Real-Time Polymerase Chain Reaction (qRT−PCR)

TRIzol reagent (Takara Biotechnology Co., Ltd., Dalian, China) was utilized to isolate total RNA from fresh CCA and paracancerous tissues and 6 cell lines. SYBR-Green Mix (Yeasen, Shanghai, China) was used according to the manufacturer’s protocol. Primer (Sangon Biotech, China) information is shown in **Supplementary Table S1**. B-ACTIN was utilized as an internal control. The 2-ΔΔCt method was applied to calculate the relative expression levels of genes.

### Immunohistochemistry

Fresh tissue samples of 7 CCA patients were obtained from Xinhua Hospital Affiliated with Shanghai Jiao Tong University School of Medicine. Seven pairs of CCA tissues and paracancerous tissues were embedded in paraffin blocks. After cutting the paraffin block into 5 micron slices, it was dewaxed with xylene and rehydrated with different concentration gradients of ethanol. The slides were then immersed in citric acid and boiled to retrieve the antigen. Subsequently, slides were incubated with a 1:100 dilution of antibodies against ATP7A (Sabbiotech), FDX1 (Proteintech), DBT (Bioss) and LIAS (Proteintech) for 1 hour at room temperature. Slides were rinsed with PBS and incubated with secondary antibody (1:100) for 30 minutes at room temperature. Slices were stained with diaminobenzidine tetrahydrochloride (DAB) counterstained with hematoxylin, dehydrated, and then sealed with neutral gum. Finally, the slides were photographed with a Leica imaging system (Wetzlar, Germany)

### Western blot

Cells were washed with PBS and lysed with Radioimmunoprecipitation assay (RIPA) buffer. Then the samples were centrifuged at 14,000×g for 15 min at 4°C in a suitable centrifuge tube. Total protein samples were separated on a 15% or 10% SDS-PAGE gel and transferred onto PVDF membrane. The PVDF membrane was blocked with 5% nonfat dry milk in TBST for an hour. Then the PVDF membrane was incubated with primary antibody overnight at 4°C (DLAT: Proteintech, 13426-1-AP. LIAS: Proteintech, 11577-1-AP. FDX1: Proteintech,12592-1-AP. GAPDH: Proteintech, 10494-1-AP) (all diluted 1:2500). After 3 times wash, the PVDF membrane was incubated with horseradish peroxidase (HRP)-conjugated secondary antibody for an hour in room temperature. Then the PVDF membrane was developed with ECL solution and photographed after 3 times-wash.

### Cell viability assay

As described in a previous study, 2500 cells/well were plated in advance in 96-well plates (5). After 16-24 hours, the indicated concentrations of the compounds elesclomol (MedChemExpress) and CuCl2 (Sigma) were added to each well (at least 3 replicate wells). We used a cell counting kit-8 (CCK-8) system to measure cell viability. Two hours after the addition of CCK-8 reagent, the optical density (OD) of each well was detected at 450 nm.

### Statistical analysis

All statistical analyses were performed in R 4.1.2. Student’s t test and ANOVA were applied to analyze the differences in continuous variables in different groups. Univariate and multivariate Cox regression analyses were utilized to identify prognosis-related CRGs. If not specified, differences were considered statistically significant at P < 0.05.

## Results

### Identification of CRGs in CCA

**Figure 1:**
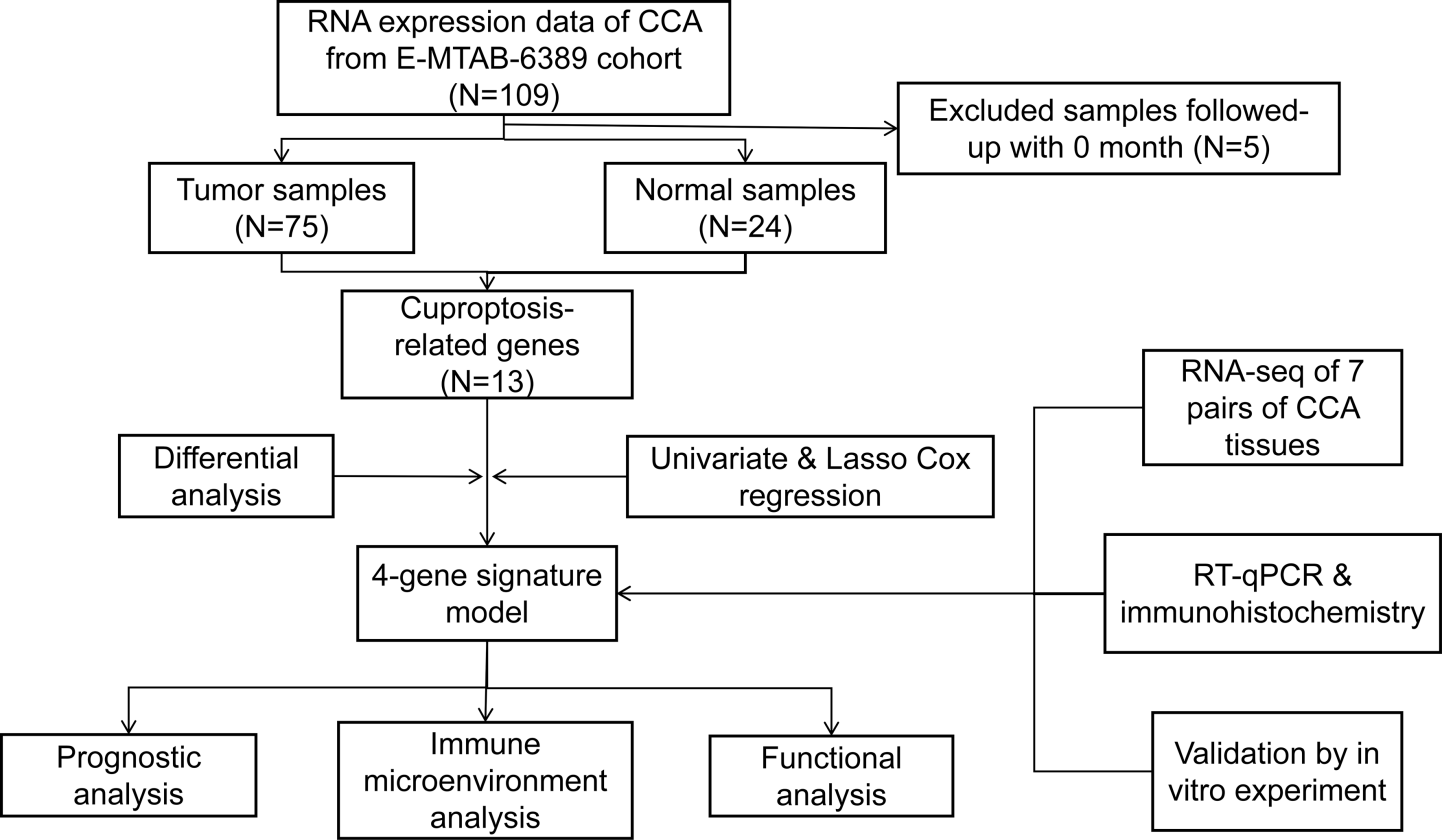
Flowchart of the entire analytical process.

**Figure 2:**
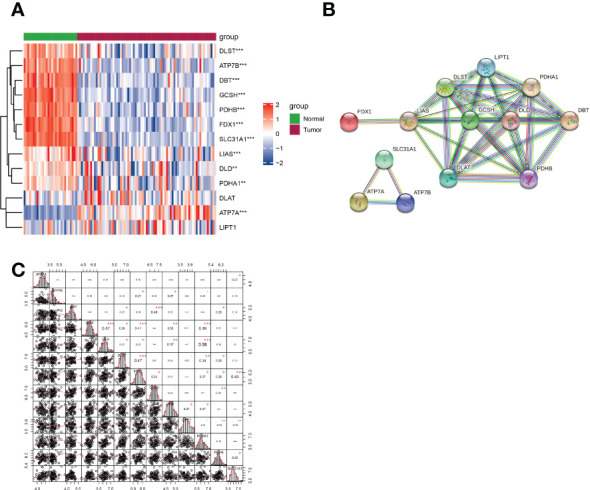
Expression of CRGs and interactions among them. **(A)** Heatmap showing the expression of CRGs in CCA and normal samples (red: upregulation; blue: downregulation; P values are shown as *p < 0.05, **p < 0.01, ***p < 0.001). **(B)** PPI network demonstrating the interactions of the CRGs (average local clustering coefficient=0.962). **(C)** The correlation plot of the CRGs.

A total of 75 patients with CCA were enrolled in this study. The detailed process is shown in **Figure 1**. Thirteen genes strongly linked to cuproptosis (ATP7A, ATP7B, FDX1, LIPT1, LIAS, DLD, DBT, GCSH, DLST, DLAT, PDHA1, PDHB, SLC31A1) were identified in previous research and literature (7). Comparing the expression levels of 13 CRGs in 29 normal and 75 tumor tissues, we identified 11 differentially expressed genes (**Figure 2A**). Among them, the differences were statistically significant for all genes except DLAT and LIPT1. A protein–protein interaction (PPI) analysis was conducted to further explore the interactions of these CRGs (**Figure 2B**). We can see that there is a close interaction between lipoylation-related proteins and Fe-S cluster proteins. In addition, three copper ion transport-related proteins interact with each other closely. The correlation between these genes is shown in **Figure 2C**. There was a significant correlation among expression levels of most CRGs.

### Construction of a CRG-based prognostic model based on the E-MTAB-6389 cohort

**Figure 3:**
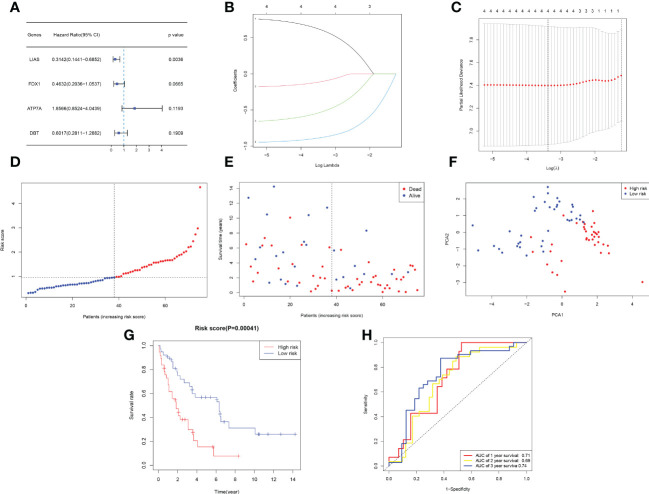
Development of a prognostic model based on the E-MTAB-6389 cohort. **(A)** Forest plot demonstrating prognosis-related genes by univariate Cox analysis. **(B–C)** A prognostic signature consisting of 4 genes was constructed through LASSO Cox regression. **(D)** Distribution of 109 patients based on the risk score (red: high risk, blue: low risk; the dotted line represents the median value). **(E)** The survival status of each patient (left side: low-risk group; right side: high-risk group). **(F)** PCA plot based on the risk score. **(G)** Kaplan−Meier survival curve according to the risk score of patients in the E-MTAB-6389 cohort. **(H)** ROC curves at 1, 3, and 5 years to evaluate the sensitivity and specificity of the risk score.

After combining gene expression and clinical information, a total of 75 CCA samples were matched with complete survival information. Primary screening of prognosis-associated genes by univariate Cox regression analysis identified four prognosis-associated candidate CRGs (P < 0.2) (**Figure 3A**). A 4-gene signature consisting of ATP7A, FDX1, DBT and LIAS was constructed by further performing LASSO regression analysis (**Figures 3B–C**). The risk score formula was as follows: Risk score = 0.7958*ATP7A+(-0.1946) *DBT+(-0.6824) *FDX1+(-0.9715) *LIAS. The risk scores of patients in the E-MTAB-6389 cohort were calculated and ranked according to the above formula, and 75 patients were divided into low- and high-risk groups (**Figure 3D**). Compared to those in low-risk group, patients in the high-risk group died more often and had shorter survival times (right side of dashed line) (**Figure 3E**). PCA revealed that patients were well divided into two clusters (**Figure 3F**). Survival analysis showed a significant difference in OS between the high- and low-risk groups (P < 0.001) (**Figure 3G**). Applying time-dependent ROC analysis to assess the sensitivity and specificity of the risk model, we discovered that the area under the ROC curve (AUC) for 1-, 2-, and 3-year survival was 0.71, 0.69, and 0.74, respectively (**Figure 3H**).

### Independent prognostic analysis of the 4-gene signature

**Figure 4:**
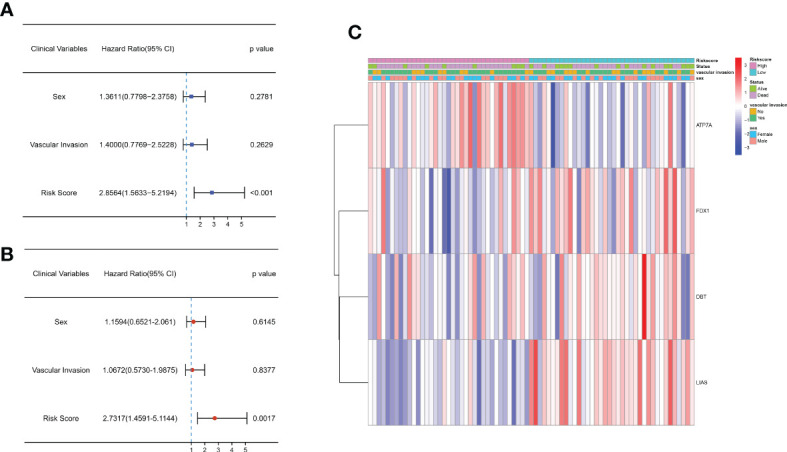
Univariate and multivariate Cox regression analyses of OS in the E-MTAB-6389 cohort. **(A)** Forest plot to demonstrate the univariate Cox regression analyses regarding OS in the E-MTAB-6389 cohort. **(B)** Forest plot showing the multivariate Cox regression analyses regarding OS in the E-MTAB-6389 cohort. **(C)** Heatmap demonstrating the correlation between 4 OS-related genes and clinical features (red: high expression; green: low expression).

Univariate and multifactorial Cox regression analyses were applied to assess whether the risk scores could be utilized as independent prognostic factors. Univariate Cox regression analysis demonstrated that the risk score was an independent predictor of poor survival in both groups (HR = 2.856, 95% CI: 1.563-5.219) (**Figure 4A**). Multivariate analysis also showed that the risk score remained a prognostic factor for both groups of CCA patients after adjusting for other confounders (HR = 2.731, 95% CI: 1.459-5.114) (**Figure 4B**). In addition, heatmaps of clinical characteristics demonstrated the distribution of the survival status of patients in both subgroups (**Figure 4C**).

### Functional analysis between the two risk groups

**Figure 5:**
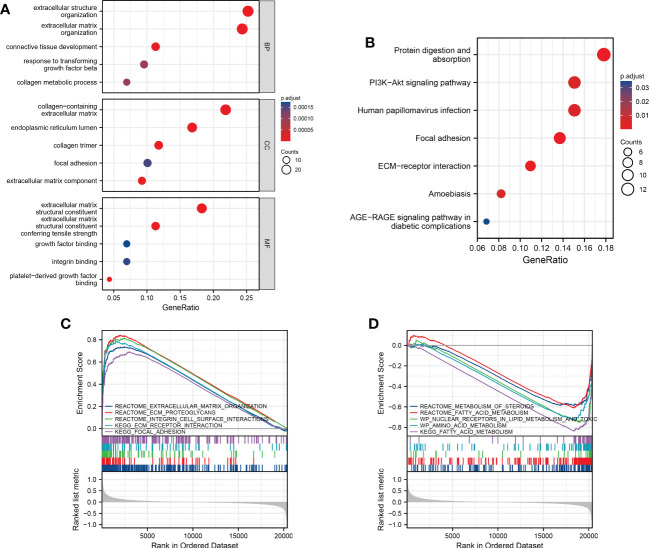
Functional analysis and the enriched immune cells between the high- and low-risk groups. **(A, B)** GO and KEGG analysis between different risk groups (BP, biological processes; CC, cellular components; MF, molecular functions; the darker the red means the more significant the difference; larger bubbles mean more enriched genes). **(C, D)** GSEA to investigate the biological pathways enriched in the high- and low-risk groups.

To further analyze the potential biological functions between subgroups classified by the risk score, we applied GO and KEGG analyses. We discovered that these DEGs were mostly associated with extracellular biological processes, such as “extracellular structure organization” and “ECM-receptor interaction”, suggesting that the high-risk subgroup was more robust in extracellular interactions than the low-risk subgroup (**Figures 5A–B**). We then used GSEA to further verify the differences in molecular mechanisms between the high-risk and low-risk groups. We obtained several enrichment results that are consistent with previous GO and KEGG analyses. The high-risk group had significantly stronger extracellular activity than the low-risk group, such as “extracellular matrix organization”, “integrin cell surface interactions” and “focal adhesion”. While metabolic activity was significantly stronger in the low-risk group, metabolic activity was significantly stronger in the low-risk group, which seems to be consistent with the metabolic mechanism of cuproptosis. The “KEGG fatty acid metabolism” pathway, “wp nuclear receptors in lipid metabolism and toxicity” and “reactome metabolism of steroids” results suggest that cuproptosis is inextricably linked to lipid metabolism (**Figures 5C, D**).

### Correlation between the risk score and immune cell infiltration

**Figure 6:**
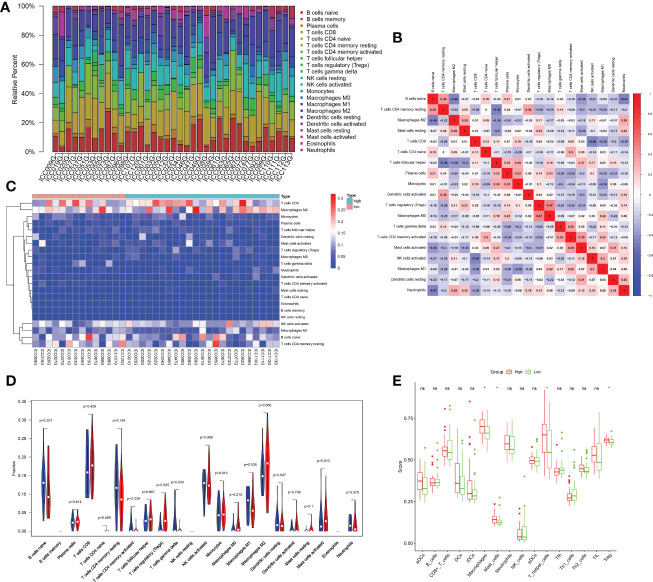
Analysis of the immune microenvironment. **(A)** Bar plot showing the proportion of different immune cells in each sample. **(B)** The corheatmap demonstrated the correlations among different infiltrated immune cells. **(C)** Heatmap showing infiltrated immune cells in the high- and low-risk groups. **(D)** Comparison of immune cell infiltration scores between the low- and high-risk groups estimated by CIBERSORT (red: high risk; blue: low risk). **(E)** Comparison of immune cell infiltration scores between the low- and high-risk groups estimated by ssGSEA (red: high risk; green: low risk). ; *p < 0.05; ns p > 0.05.

To better understand whether the stronger extracellular activity in the high-risk group was related to the immune microenvironment, we performed CIBERSORT analysis to assess immune cell infiltration. T cells and macrophages accounted for the majority of infiltrated immune cells in different samples. Among them, CD8+ T cells and M2 macrophages were the most predominant cell subtypes (**Figure 6A**). There was also a link between the infiltration profiles of different types of immune cells. For example, there was a correlation between Treg infiltration and M0 macrophage infiltration (**Figure 6B**). There were significant differences in immune cell infiltration between patients in the high- and low-risk groups (**Figures 6C–E**). The results based on CIBERSORT showed that Tregs were significantly abundant in the high-risk group, while activated CD4+ memory T cells and gamma delta T cells were more abundant in those in the low-risk group (**Figure 6D**). We further applied the ssGSEA algorithm to assess the difference in immune cell infiltration between the high- and low-risk groups. The results based on ssGSEA showed that macrophage, T-helper cell and Treg infiltration was significantly higher in the high-risk group than in the low-risk group (**Figure 6E**).

### Expression levels of 4 CRGs in paired tumor tissues and cell lines

**Figure 7:**
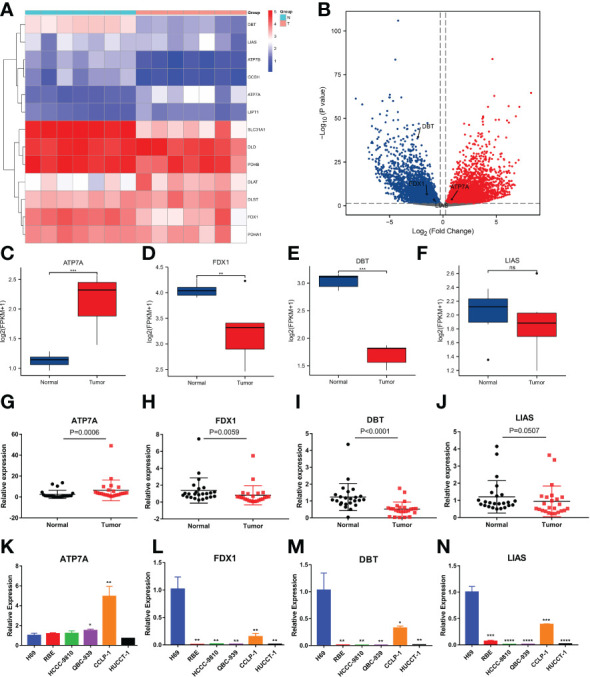
Evaluation of the expression of 4 CRGs in tissue samples and different cell types by RNA-seq and qRT−PCR. **(A, B)** Heatmap and volcano plot demonstrating CRG expression in 7 pairs of samples *via* RNA-seq. **(C–F)** Boxplots showing the expression of 4 CRGs in the sequencing results (FPKM: fragments per kilobase million). **(G-J)** qRT−PCR analysis of ATP7A, FDX1, DBT and LIAS mRNA levels in tissue samples. **(K-N)** Expression levels of 4 CRGs in the normal bile duct epithelial cells H69 and 5 types of CCA cells (RBE, HCCC-9810, QBC-939, CCLP-1 and HUCCT-1) by qRT−PCR. (∗p < 0.05, ∗∗p < 0.01, ∗∗∗p < 0.001, ∗∗∗∗p< 0.0001 and ns p > 0.05).

**Figure 8:**
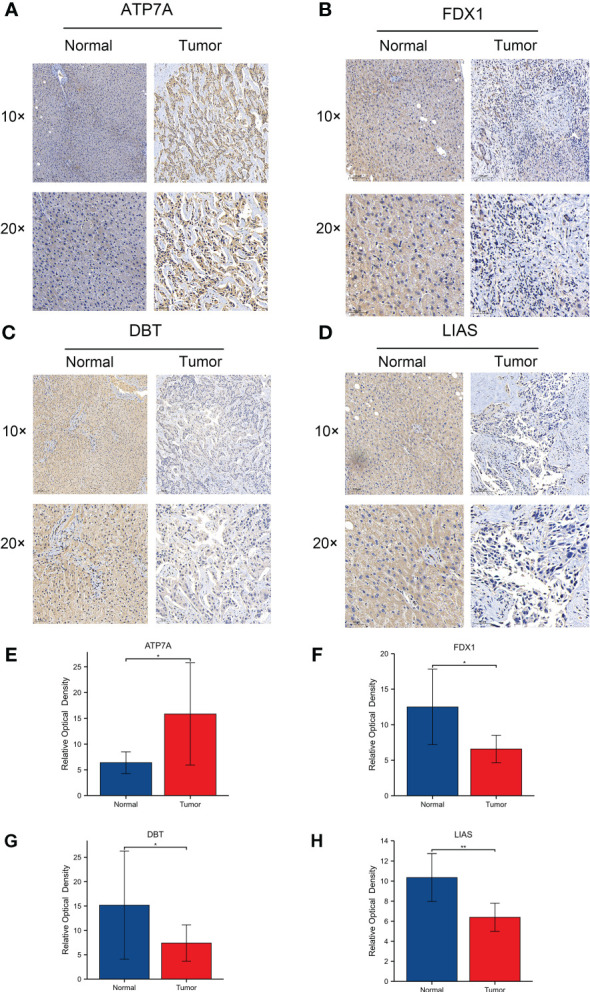
**(A-D)** Protein expression levels of 4 CRGs using immunohistochemistry. 1054063 w/ supp The expression levels of ATP7A, FDX1, DBT and LIAS in adjacent normal tissues and tumor tissues. **(E-H)** Relative optical densities of the 4 genes. (∗p < 0.05, ∗∗p < 0.01).

Next, we performed RNA sequencing on seven pairs of freshly resected CCA tissues. The sequencing results were generally consistent with the dataset results (**Figures 7A, B**). The FPKM values of the 4 genes showed that the fold changes in the expression levels of ATP7A, FDX1 and DBT were significantly different, but the downregulation of LIAS was not statistically significant (**Figures 7C–F**). To further validate the expression levels of the four key genes in CCA, we additionally collected twenty-four pairs of CCA tissues. The qRT−PCR results showed that the expression levels of FDX1, DBT were significantly lower than those in normal tissues. The expression level of LIAS in tumor tissues was lower than those in normal tissues but the P value was not significant (P=0.0507). The ATP7A expression level was significantly higher than that in normal tissues (**Figures 7G–J**). The expression levels of the 4 genes in various types of cells (RBE, HCCC-9810, QBC-939, CCLP-1, and HUCCT-1) were basically consistent with the dataset (**Figures 7K–N**). Immunohistochemical results showed that the 4 genes were mainly expressed in the cytoplasm (**Figures 8A–D**). ATP7A expression was significantly higher in tumor tissues than in paraneoplastic tissues, while FDX1, DBT, and LIAS expression was significantly lower than in paraneoplastic tissues (**Figures 8E–H**) (P<0.05).

### In vitro validation of the key gene in the 4-gene signature

**Figure 9:**
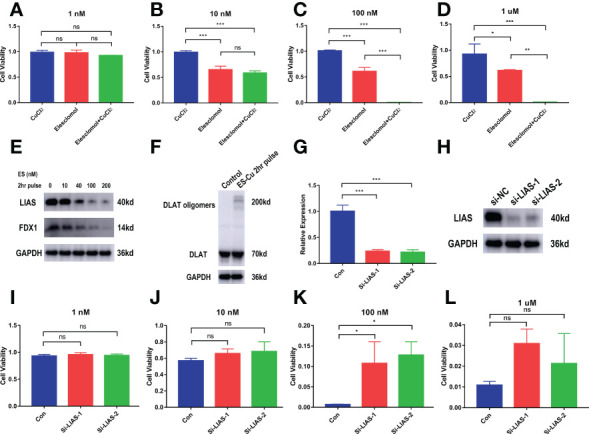
*In vitro* validation of the effect of cuproptosis on CCA and the role of the key gene LIAS. **(A–D)** CCK-8 assay demonstrating the viability of cells treated with the indicated concentration of CuCl2 or elesclomol alone or in combination for 72 h (n = 3). **(E)** CCLP-1 cells were analyzed for indicated proteins 24 hours after pulse treatment (2 hours) with indicated concentrations of elesclomo (media supplemented with 1µM CuCl2) (ES, elesclomo) **(F)** DLAT oligomerization level was detected after 2-hour pulse treatment. **(G–H)** The knockdown efficiency of LIAS. **(I-L)** LIAS knockdown attenuates the toxicity of cuproptosis in the CCA cells CCLP-1 (Con, LIAS knockdown negative control. Si-LIAS, LIAS knockdown). (∗p < 0.05, ∗∗p < 0.01, ∗∗∗p < 0.001, and ns p > 0.05).

We further examined the effect of cuproptosis on CCA cell lines. To clarify whether ES-Cu can induce cuproptosis in CCA cells, we used different concentrations of the compounds to stimulate the cells (CCLP-1) for 72 hours. The simultaneous addition of elesclomol and Cu2+ produced greater toxicity to the cells than the addition of elesclomol alone. However, the addition of CuCl2 alone hardly affected the cell viability (**Figures 9A–D**). After pulse treatment with elesclomol and Cu2+ for 2 hours, we detected the expression levels of Fe-S cluster proteins 24 hours later and found that FDX1 and LIAS gradually decreased in protein level with increasing concentration (**Figure 9E**). DLAT oligomerization level increased after the pulse treatment (**Figure 9F**). This evidence suggests that cuproptosis occurs in CCA cells in the presence of ES-Cu. Next, we analyzed the potential functions of LIAS in cuproptosis in CCA. In the case of proper knockdown efficiency (**Figures 9G–H**), silencing LIAS significantly attenuated the damaging effects of elesclomol and Cu2+ on CCA cell viability (**Figures 9I–L**).

## Discussion

CCA is a highly heterogeneous malignancy, and its overall incidence is on the rise globally. Radical surgery is the only potentially curative treatment option (19). However, because of its insidious onset, CCA patients are often diagnosed when surgery is no longer an option (20). Therefore, the selection of appropriate biomarkers to predict patient prognosis is crucial. Programmed death is one of the most promising breakthroughs in the treatment of malignancies (21, 22). Exploring new mechanisms of programmed death in CCA is critical. Cuproptosis is a newly identified pattern of programmed death (7, 23). It is essential to study its association with the prognosis of CCA and to explore its impact on the progression of tumor cells.

As a newly discovered type of programmed cell death, cuproptosis offers new ideas for cancer treatment. Cuproptosis leads to proteotoxic stress through excess intracellular copper-induced aggregation of lipoylated dihydrolipoamide S-acetyltransferase (DLAT). This copper-induced death is closely related to the mitochondrial TCA cycle. In recent years, an increasing number of studies have focused on targeting programmed cell death (24, 25). Cuproptosis as a therapeutic target in CCA is not yet clear.

In the present study, we first obtained the mRNA levels of 13 genes closely associated with cuproptosis in 75 tumor samples. A 4-gene risk signature was then constructed by Cox univariate analysis and LASSO regression analysis. Functional analysis suggested that extracellular signaling activity was significantly enhanced in the high-risk group, while metabolic activity was significantly enhanced in the low-risk group. This result is consistent with the mechanism by which cuproptosis depends on lipid metabolism. Lipid metabolism has been shown to be associated with malignant progression in a variety of tumors, including CCA (26–28). However, the link between lipid metabolism and cuproptosis needs to be further explored. In contrast, the enhanced extracellular signaling activity in the high-risk group is likely to be closely related to immunity. Immune cell infiltration was compared between the low-risk and high-risk groups, and the level of immune cell infiltration, including macrophages and Tregs, was found to be elevated in the high-risk group compared to the low-risk group. An increase in tumor-associated macrophages and Treg cells can create a suppressive immune microenvironment that is associated with poor patient prognosis (29–31). Treg infiltration was increased in tumor tissues of patients with CCA, suggesting that it may be related to the development of CCA (32). A higher percentage of Tregs tends to represent poorer immunotherapeutic outcomes. The formation of a suppressive immune microenvironment in high-risk patients may be one of the reasons for their poor prognosis. Further studies are needed to reveal how cuproptosis reshapes the immune microenvironment in CCA patients.

The prognostic model proposed in this study consists of 4 CRGs (LIAS, FDX1, DBT, ATP7A). Lipoic acid synthase (LIAS) catalyzes the final step of lipoic acid synthesis (33). LIAS belongs to the lipoic acid synthase family and is localized in mitochondria. Defective expression of LIAS has been detected in diabetes, atherosclerosis and other diseases (34, 35). However, there are no reports of LIAS in cancer research. Our study found that LIAS can be an independent prognostic factor in CCA patients. Moreover, knockdown of LIAS significantly improved the tolerance of CCA cells to cuproptosis. Whether LIAS can be further targeted in CCA patients remains to be confirmed by further studies. Ferredoxin 1 (FDX1), which encodes a small iron-sulfur protein, has been shown to contribute to the accumulation of toxic lipid acylated DLAT (36). In our study, the expression level of FDX1 in CCA was significantly downregulated. ATP7A is a key transporter of copper efflux. Under conditions of elevated extracellular copper ions, it relocalizes from the Golgi apparatus to the plasma membrane and promotes copper ion excretion from the cell (37). ATP7A was found to be upregulated in multiple cancers and associated with cancer progression. ATP7A promotes tumor growth and metastasis in lung and breast cancers (38, 39). ATP7A is upregulated in gemcitabine-resistant non-small cell lung cancer (40). In esophageal and ovarian cancers, ATP7A is associated with cisplatin resistance (41–43). Our study demonstrated for the first time that ATP7A is upregulated in CCA; however, whether it promotes CCA progression needs to be confirmed by further studies. Dihydrolipoamide branched chain transacylase E2 (DBT) regulates the metabolic complex of carbon entry points of the TCA cycle (44). DBT is also one of the four enzymes that can undergo lipid acylation (7). Cu2+ increased mitochondrial lipid acylation, one of the critical aspects of cuproptosis. Our study identified significantly low expression of DBT for the first time in CCA tissues and cell lines, and its specific mechanism in cuproptosis in CCA needs to be further explored.

We further validated the expression of these 4 genes in multiple ways. Immunohistochemical results showed that FDX1, DBT and LIAS expression were significantly downregulated and ATP7A expression was significantly upregulated in CCA tissues. This is consistent with the results of the E-MTAB-6389 dataset. qRT−PCR results on CCA samples showed that FDX1, DBT were significantly downregulated in tumor tissue samples, while ATP7A was significantly upregulated. The expression level of LIAS in tumor tissues was lower than those in normal tissues but the difference was not statistically significant (P=0.0507). The RNA-seq results showed no significant difference in LIAS expression, while the expression levels of the other three genes were consistent with the dataset results. We speculate that this may be related to the small number of samples tested. LIAS was identified as an independent prognostic factor in the multivariate Cox regression analysis. We then focused on verifying the role of LIAS in the occurrence of cuproptosis in CCA in vitro. On the premise that ES+Cu can effectively induce cuproptosis in CCA cell lines, we knocked down the expression of LIAS. The results were consistent with previous studies showing that knockdown of LIAS attenuated the toxic effects of cuproptosis on CCA cells. These results suggest that LIAS plays an important role in the occurrence of cuproptosis in CCA.

Our research has some limitations. First, the prognostic model was only validated in the E-MATB-6389 cohort because of the paucity of available data on CCA. Therefore, further external validation in different CCA cohorts with larger sample sizes is needed. Second, the cell lines selected for in vitro experimental validation were limited. The results may be biased by cell characteristics and cell status. Third, expression validation using patient tissues was low and further larger numbers of samples are needed for expression validation to obtain more accurate results. When using patient tissues for expression validation, the number of tissues was small, and more samples are needed to obtain more reliable results.

## Conclusion

A novel cuproptosis-related model for predicting the prognosis of CCA patients was constructed and systematically analyzed in this study. The risk score based on 4 CRGs was an independent risk factor for predicting OS. This study is the first to explore the biological role of cuproptosis in CCA. We also found that cuproptosis was associated with altered cellular metabolic activity and immune microenvironment. We hope that the model will provide a reference for predicting the survival of CCA patients and guiding the related treatment of CCA patients.

## Data availability statement

Publicly available datasets were analyzed in this study. This data can be found here: https://www.ebi.ac.uk/biostudies/arrayexpress/studies/E-MTAB-6389.

## Ethics statement

The studies involving human participants were reviewed and approved by The Ethics Committee of Xinhua Hospital Affiliated to Shanghai Jiao Tong University School of Medicine. The patients/participants provided their written informed consent to participate in this study. Written informed consent was obtained from the individual(s) for the publication of any potentially identifiable images or data included in this article.

## Author contributions

JC completed the construction of the model and the writing of the manuscript. XY, HT, and CT completed critical review and guidance of the manuscript. ZT completed model guidance, critical review, and funding support. All authors read and approved the final manuscript.
