# Supplementary material for: Establishment and experimental validation of a novel cuproptosis-related gene signature for prognostic implication in cholangiocarcinoma
Source: Front Oncol. 2022 Dec 8;12:1054063. doi: 10.3389/fonc.2022.1054063 (PMC9773249; doi:10.3389/fonc.2022.1054063)
Supplement: Supplementary file 1 [file DataSheet_1.zip › Supplementary Material/S1.Primers.docx]

| Primers | Forward primer (5′-3′) | Reverse primer (5′-3′) |
| --- | --- | --- |
| ATP7A | CACCTTGTTTCTGACTGTTACG | CCAGCTCTTTTATCTGATTGGC |
| FDX1 | TCGATCTGGCATATGGACTAAC | GCACTCGAACAGTCATATTGTC |
| DBT | AATGGTCAAGACTATGTCTGCA | AATTCTTCTCGGAGCTTAACCA |
| LIAS | GTATGTGAGGAAGCTCGATGTC | CACCCATCAACATGATCGTGG |
